# Supplementary material for: Brain-enriched RagB isoforms regulate the dynamics of mTORC1 activity through GATOR1 inhibition
Source: Nat Cell Biol. 2022 Sep 12;24(9):1407–21. doi: 10.1038/s41556-022-00977-x (PMC9481464; doi:10.1038/s41556-022-00977-x)
Supplement: Supplementary file 1 — Reporting Summary [file 41556_2022_977_MOESM1_ESM.pdf]

nature portfolio

Corresponding author(s): Aurelio Teleman

Last updated by author(s): Jun 19, 2022

Reporting Summary

Nature Portfolio wishes to improve the reproducibility of the work that we publish. This form provides structure for consistency and transparency in reporting. For further information on Nature Portfolio policies, see our Editorial Policies and the Editorial Policy Checklist.

Statistics

For all statistical analyses, confirm that the following items are present in the figure legend, table legend, main text, or Methods section.

n/a	Confirmed	
☐	☒	The exact sample size (n) for each experimental group/condition, given as a discrete number and unit of measurement
☐	☒	A statement on whether measurements were taken from distinct samples or whether the same sample was measured repeatedly
☐	☒	The statistical test(s) used AND whether they are one- or two-sided<br>*Only common tests should be described solely by name; describe more complex techniques in the Methods section.*
☒	☐	A description of all covariates tested
☐	☒	A description of any assumptions or corrections, such as tests of normality and adjustment for multiple comparisons
☐	☒	A full description of the statistical parameters including central tendency (e.g. means) or other basic estimates (e.g. regression coefficient) AND variation (e.g. standard deviation) or associated estimates of uncertainty (e.g. confidence intervals)
☐	☒	For null hypothesis testing, the test statistic (e.g. F, t, r) with confidence intervals, effect sizes, degrees of freedom and P value noted<br>*Give P values as exact values whenever suitable.*
☒	☐	For Bayesian analysis, information on the choice of priors and Markov chain Monte Carlo settings
☒	☐	For hierarchical and complex designs, identification of the appropriate level for tests and full reporting of outcomes
☒	☐	Estimates of effect sizes (e.g. Cohen's d, Pearson's r), indicating how they were calculated

Our web collection on statistics for biologists contains articles on many of the points above.

Software and code

Policy information about availability of computer code

Data collection	Xena browser (xenabrowser.net)
Data analysis	Image Lab 5.2.1<br>Microsoft Excel 16.16.27<br>CellProfiler 3.1.5<br>GraphPad Prism 9.0.2<br>Xena browser (xenabrowser.net)<br>FlowJo (v10)

For manuscripts utilizing custom algorithms or software that are central to the research but not yet described in published literature, software must be made available to editors and reviewers. We strongly encourage code deposition in a community repository (e.g. GitHub). See the Nature Portfolio guidelines for submitting code & software for further information.

Data

Policy information about availability of data

All manuscripts must include a data availability statement. This statement should provide the following information, where applicable:

- Accession codes, unique identifiers, or web links for publicly available datasets
- A description of any restrictions on data availability
- For clinical datasets or third party data, please ensure that the statement adheres to our policy

Source data have been provided in Source Data. Data on RagA/B expression in brain cell types were extracted from the RNAseq dataset published by the Barres

group (GEO accession number: GSE52564). Data from the GTEX and TCGA databases used in Fig. 6 and Extended Data Fig. 10 were accessed through the Xena browser (xenabrowser.net). All other data supporting the findings of this study are available from the corresponding author on reasonable request.

Field-specific reporting

Please select the one below that is the best fit for your research. If you are not sure, read the appropriate sections before making your selection.

☒ Life sciences ☐ Behavioural & social sciences ☐ Ecological, evolutionary & environmental sciences

For a reference copy of the document with all sections, see nature.com/documents/nr-reporting-summary-flat.pdf

Life sciences study design

All studies must disclose on these points even when the disclosure is negative.

Sample size	Samples sizes were chosen based on standard practice in the field (e.g. PMID: 18497260, 20381137, 26972053, 23723238, 24529379)
Data exclusions	No data were excluded.
Replication	All experiments were repeated two to three times with independent biological replicates. All attempts at replication were successful. For immunofluorescence and biochemical experiments, one representative experiment is shown.
Randomization	No randomization was used to allocate samples in experiments comparing different cell lines. When comparing different treatments within the same cell line, samples were assigned randomly to control or treatment group.
Blinding	No blinding was used since experiments required frequent intervention by investigators to maintain cell lines, thus precluding effective blinding.

Reporting for specific materials, systems and methods

We require information from authors about some types of materials, experimental systems and methods used in many studies. Here, indicate whether each material, system or method listed is relevant to your study. If you are not sure if a list item applies to your research, read the appropriate section before selecting a response.

Materials & experimental systems

n/a	Involved in the study	
☐	☒	Antibodies
☐	☒	Eukaryotic cell lines
☒	☐	Palaeontology and archaeology
☐	☒	Animals and other organisms
☒	☐	Human research participants
☒	☐	Clinical data
☒	☐	Dual use research of concern

Methods

n/a	Involved in the study	
☒	☐	ChIP-seq
☐	☒	Flow cytometry
☒	☐	MRI-based neuroimaging

Antibodies

Antibodies used

The following primary antibodies were used:
RagA (recognizing also both RagB isoforms) (Cell Signaling Technology #4357)
RagC (Cell Signaling Technology #9480)
RagD (Cell Signaling Technology #4470)
phospho-S6K1 T389 (Cell Signaling Technology #9205)
S6K1 (Cell Signaling Technology #2708)
phospho-TFEB S211 (Cell Signaling Technology #37681)
TFEB (Cell Signaling Technology #37785)
phospho-4EBP1 S65 (Cell Signaling Technology #9451)
4EBP1 (Cell Signaling Technology #9452)
FLAG tag (Sigma-Aldrich #F7425, 1:2500)
alpha-tubulin (Sigma-Aldrich #T9026, 1:5000)
mTOR (Cell Signaling Technology #2983, 1:200 for immunofluorescence, 1:1000 for western blots)
Raptor (Cell Signaling Technology #2280)
LAMP2 (Hybridoma Bank #H4B4, 1:50 for immunofluorescence, 1:500 for western blots)
calreticulin (Cell Signaling Technology #12238)
VDAC (Cell Signaling Technology #4661)
HA tag (Cell Signaling Technology #2367, used to detect HA-tagged proteins in whole cell lysates)
HA tag (Cell Signaling Technology #3724, used to detect HA-tagged proteins in immunoprecipitates)
LAMTOR1/p18 (Cell Signaling Technology #8975)

GAPDH (Cell Signaling Technology #2118)
myc tag (Cell Signaling Technology #2278)
TSC2 (Cell Signaling Technology #4308)
Rheb (Abnova #H00006009-M01)
DEPDC5 (Abcam #ab185565, used to screen DEPDC5KO clones)
calnexin (Enzo #ADI-SPA-960-D).

All antibodies were diluted 1:1000 unless indicated otherwise.

The following secondary antibodies were used:
anti-rabbit HRP (Jackson ImmunoResearch #111-035-003, used 1:10000)
anti-mouse HRP (Jackson ImmunoResearch #115-035-003, used 1:10000)
anti-rabbit Alexa488 (Life Technologies #A11008, used 1:500)
anti-mouse TRITC (Jackson ImmunoResearch #715-025-151, used 1:200)

Validation

RagA, RagC, RagD: validated for WB in previous publications and by this study through corresponding knockout cell lines (Extended Data Fig. 1a,d)
phospho-S6K1 T389 and S6K1: validated for WB by the manufacturer (cells treated with insulin) and by this study through treatments that affect mTORC1 activity (e.g. Fig. 1e)
phospho-TFEB S211 and TFEB: validated for WB by the manufacturer (cells treated with torin1) and by this study through treatments that affect mTORC1 activity (e.g. Fig. 1e)
phospho-4EBP1 S65 and 4EBP1: validated for WB by the manufacturer (cells treated with insulin) and by this study through treatments that affect mTORC1 activity (e.g. Fig. 1e)
FLAG tag, HA tag (#2367 and #3724), and myc tag: validated for WB by the manufacturer (cells overexpressing tagged proteins) and by this study through overexpression of tagged proteins (e.g. Fig. 2g,j)
alpha-tubulin: validated for WB by the manufacturer using independent antibody validation
mTOR: validated for immunostaining in Sancak Y et al., Science, 2008 (PMID:18497260), for WB in Sherman et al., J Neurosci, 2012 (PMID: 22302821)
Raptor: validated for WB in Rogala et al., Science, 2019 (PMID: 31601708)
LAMP2: validated for WB in Majer F, Gene, 2012 (PMID: 22365987)
calreticulin: validated for WB in Wolfson et al., Nature, 2017 (PMID: 28199306)
VDAC: validated for WB in Li et al., Sci Rep, 2018 (PMID: 29572489)
LAMTOR1/p18: validated for WB in Son et al., Cell Metab, 2019 (PMID: 30197302)
GAPDH: validated for WB in Mungrue et al., J Immunol, 2009 (PMID: 19109178)
TSC2: validated for WB by the manufacturer using a TSC2-null cell line
Rheb: validated for WB by the manufacturer using recombinant protein
DEPDC5: validated for WB in De Fusco et al., Neurobiol. Dis., 2020 (PMID: 32113911)
calnexin: validated for WB in Gardner et al., Arch Biochem Biophys, 2000 (PMID: 10871059)

Eukaryotic cell lines

Policy information about cell lines

Cell line source(s)

HEK293T: ATCC #CRL-3216
HEK293: Stratagene #240073
Neuro-2a: ATCC #CCL-131
U2OS: ATCC #HTB-96
HeLa: ATCC #CCL-2
HepG2: ATCC #HB-8065
EFO-21: DMSZ #ACC 235

Authentication

None.

Mycoplasma contamination

No mycoplasma contamination was detected by qPCR.

Commonly misidentified lines
(See ICLAC register)

None of these cell lines were used.

Animals and other organisms

Policy information about studies involving animals; ARRIVE guidelines recommended for reporting animal research

Laboratory animals

C57B6/J time-mated 2-3 months old female mice (E15): isolation of MEFs and neurons
C57B6/J 2-3 months old female mice: tissue extraction
C57BL/6NCrl P0 pups: isolation of neurons

Wild animals

No wild animals were used.

Field-collected samples

No samples were field-collected.

Ethics oversight

All animal experiments conform to the relevant regulatory standards of the German Cancer Research Center and have been approved by the ethical authorities, Regierungspräsidium Karlsruhe, Germany.

Note that full information on the approval of the study protocol must also be provided in the manuscript.

Flow Cytometry

Plots

Confirm that:

☒ The axis labels state the marker and fluorochrome used (e.g. CD4-FITC).

☒ The axis scales are clearly visible. Include numbers along axes only for bottom left plot of group (a 'group' is an analysis of identical markers).

☒ All plots are contour plots with outliers or pseudocolor plots.

☒ A numerical value for number of cells or percentage (with statistics) is provided.

Methodology

Sample preparation	20 µM OPP reagent (Jena Bioscience #NU-931-05) was added for 30 minutes to the cells. The cells were subsequently washed with DPBS, trypsinized, and fixed with ice-cold 70% ethanol for 30 minutes at -20C degrees, followed by three washes in PBS supplemented with 0.5% Tween-20. The incorporated OPP was then labeled with the Alexa488 Fluor Picolyl azide using the Click-iT Plus OPP Protein synthesis assay kit (Life Technologies #C10456), as per manufacturer's instructions.
Instrument	Guava easyCyte HT flow cytometer (Millipore)
Software	FlowJo (v10)
Cell population abundance	29.8% (87.7% singlets)
Gating strategy	The cell population of interest was identified plotting FSC-H vs SSC-H, singlets gated by plotting FSC-H vs FSC-A, and the mean intensity of the Alexa488 signal within the singlets population was used to quantify the extent of OPP incorporation.

☒ Tick this box to confirm that a figure exemplifying the gating strategy is provided in the Supplementary Information.
