# Supplementary material for: Brain-enriched RagB isoforms regulate the dynamics of mTORC1 activity through GATOR1 inhibition
Source: Nat Cell Biol. 2022 Sep 12;24(9):1407–21. doi: 10.1038/s41556-022-00977-x (PMC9481464; doi:10.1038/s41556-022-00977-x)

Fig. 2a unprocessed blots

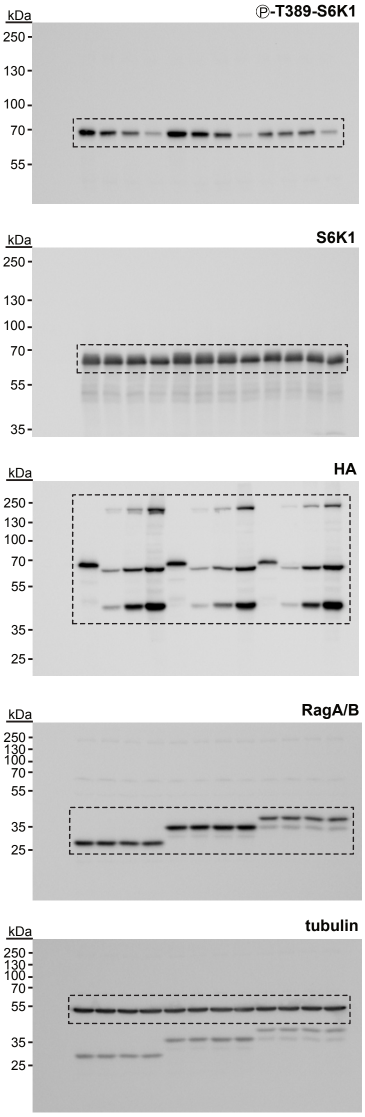

Fig. 2c unprocessed blots

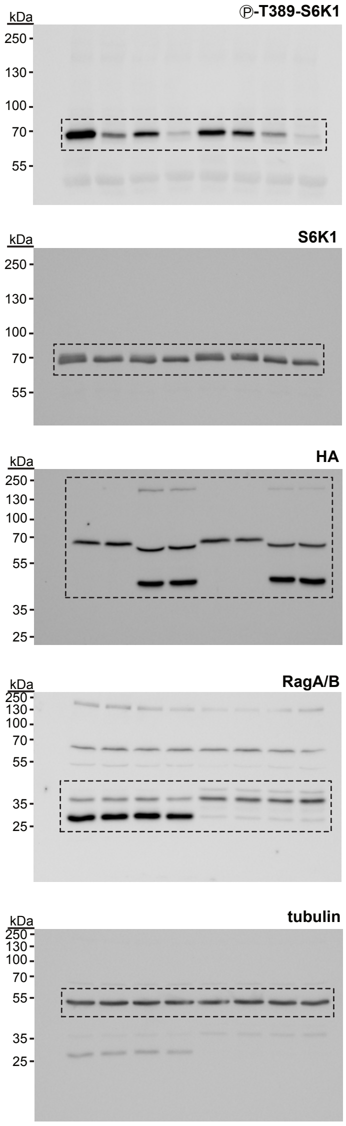

Fig. 2g unprocessed blots

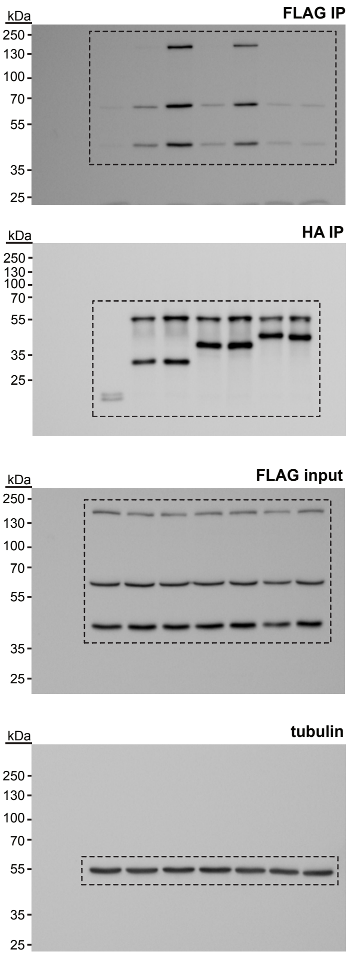

Fig. 2j unprocessed blots

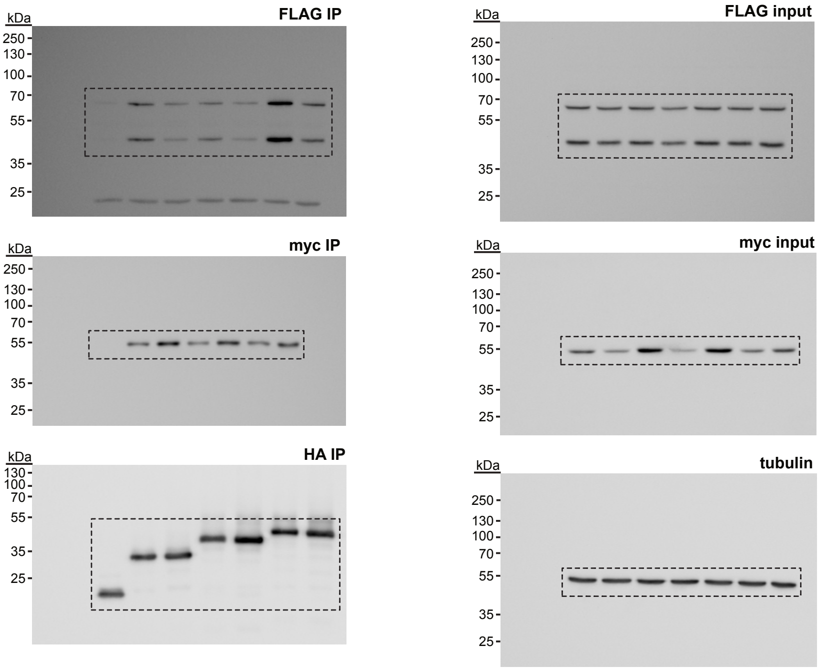

Supplement: Source Data Fig. 2 — Unprocessed western blots. [file 41556_2022_977_MOESM6_ESM.pdf]
