# Supplementary figures and images for: Brain-enriched RagB isoforms regulate the dynamics of mTORC1 activity through GATOR1 inhibition
Source: Nat Cell Biol. 2022 Sep 12;24(9):1407–21. doi: 10.1038/s41556-022-00977-x (PMC9481464; doi:10.1038/s41556-022-00977-x)

Fig. 4b unprocessed blots

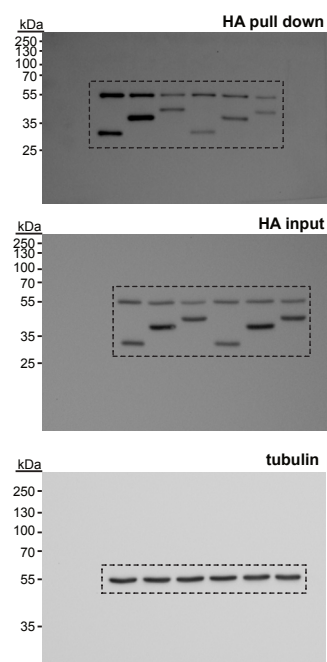

Fig. 4d unprocessed blots

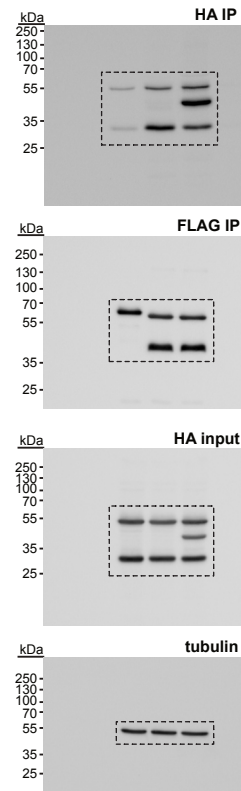

Fig. 4f unprocessed blots

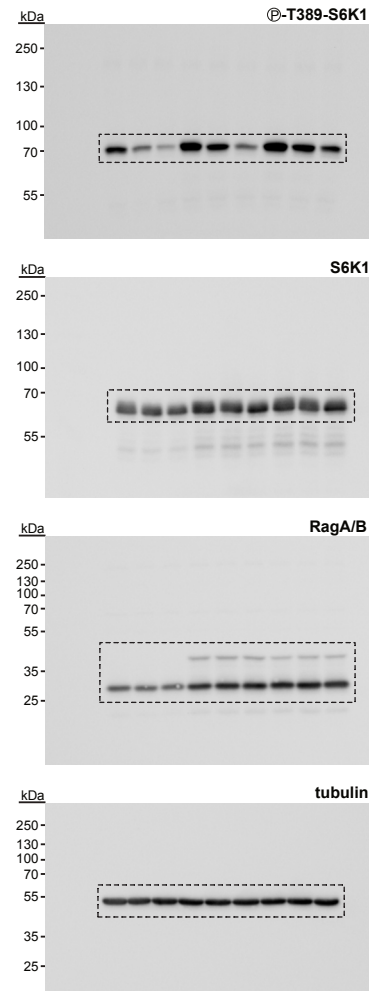

Supplement: Source Data Fig. 4 — Unprocessed Western Blots [file 41556_2022_977_MOESM10_ESM.pdf]

ED Fig. 1a unprocessed blots

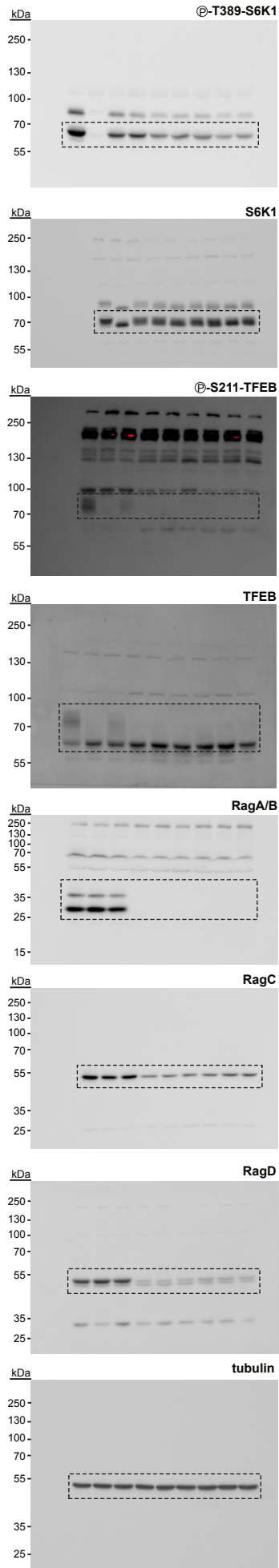

ED Fig. 1d unprocessed blots

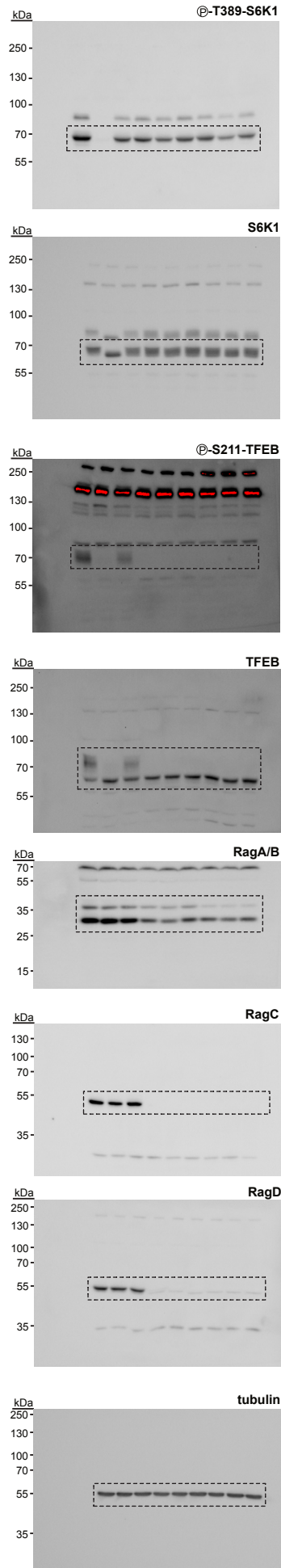

ED Fig. 1h unprocessed blots

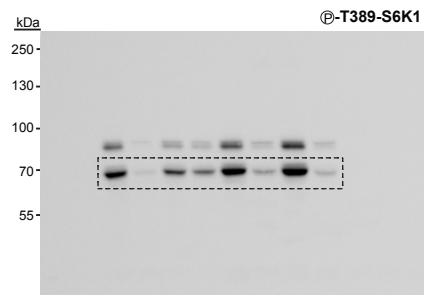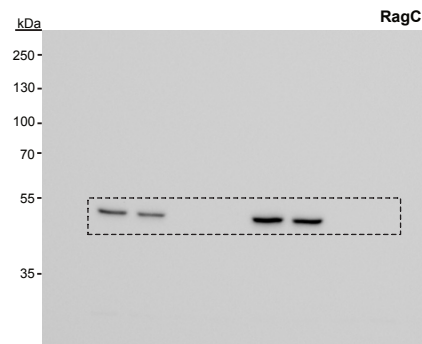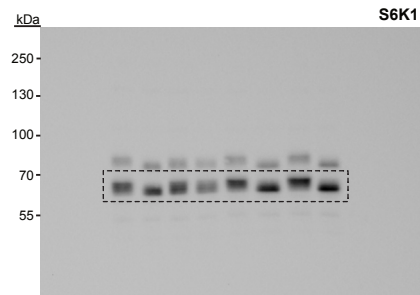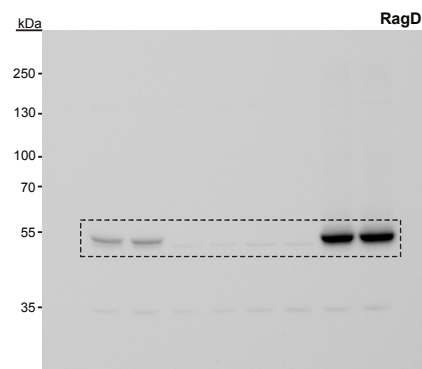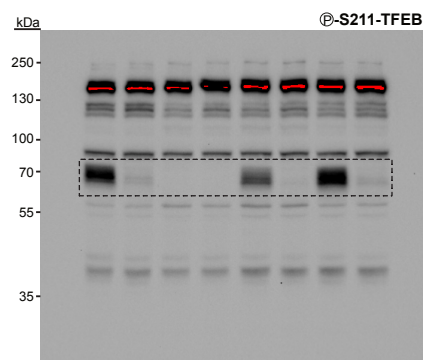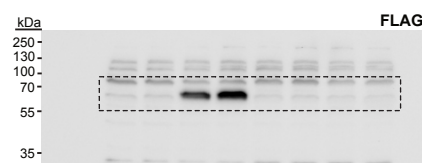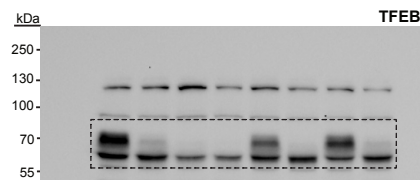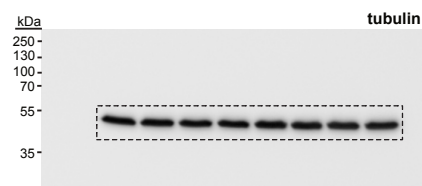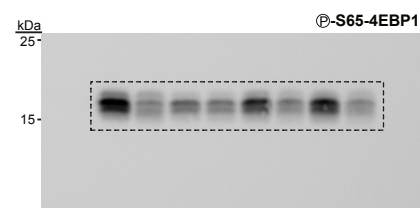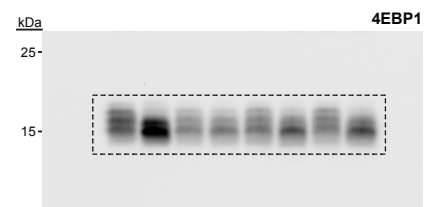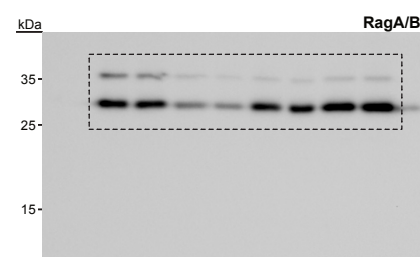

Supplement: Source Data Extended Data Fig. 1 — Unprocessed western blots. [file 41556_2022_977_MOESM16_ESM.pdf]

ED Fig. 2a unprocessed blots

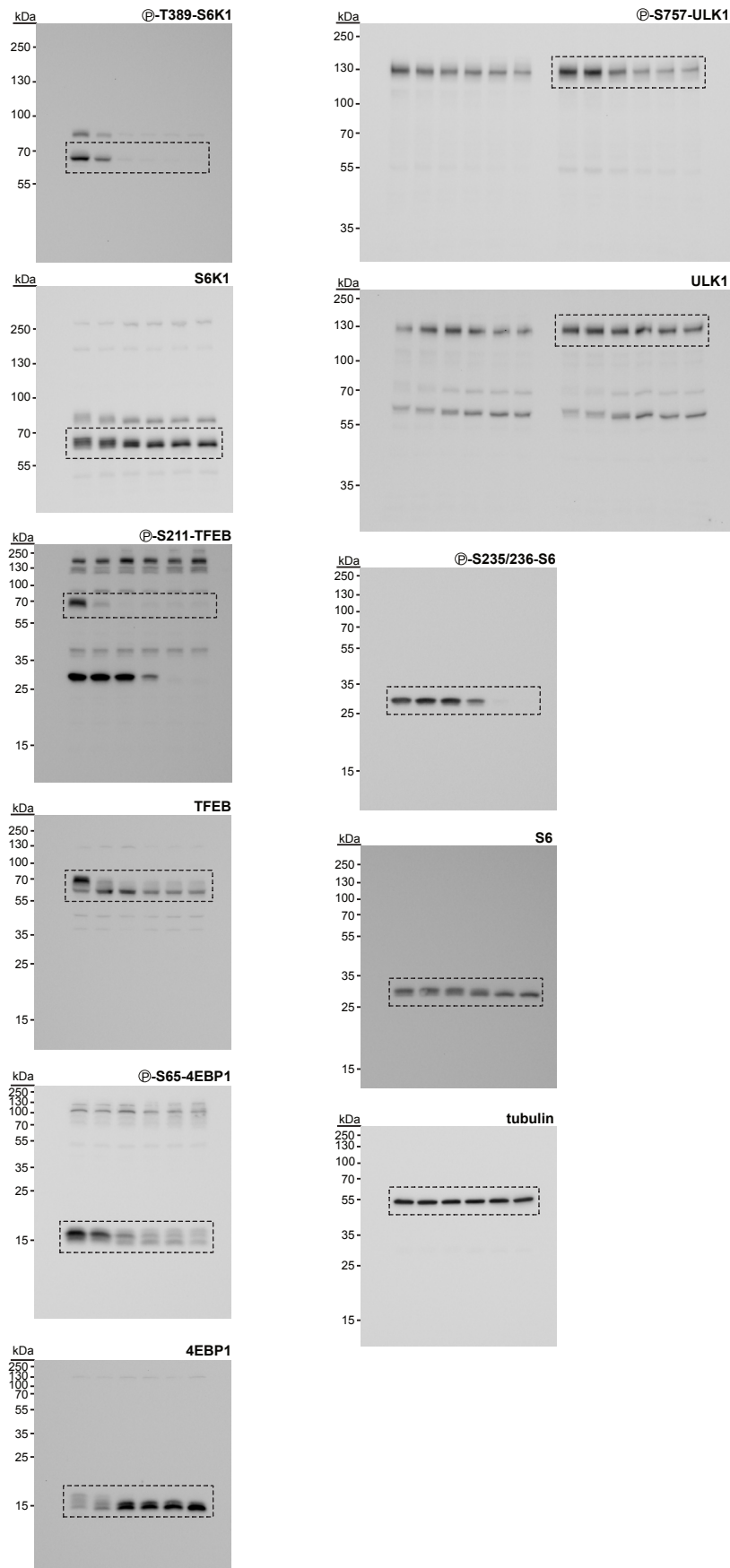

Supplement: Source Data Extended Data Fig. 2 — Unprocessed western blots. [file 41556_2022_977_MOESM18_ESM.pdf]

ED Fig. 3e unprocessed blots

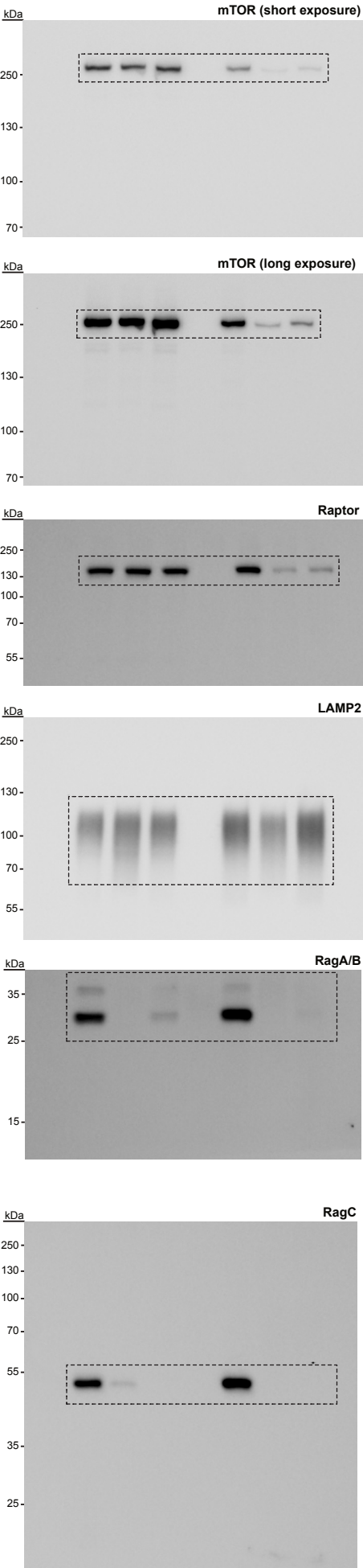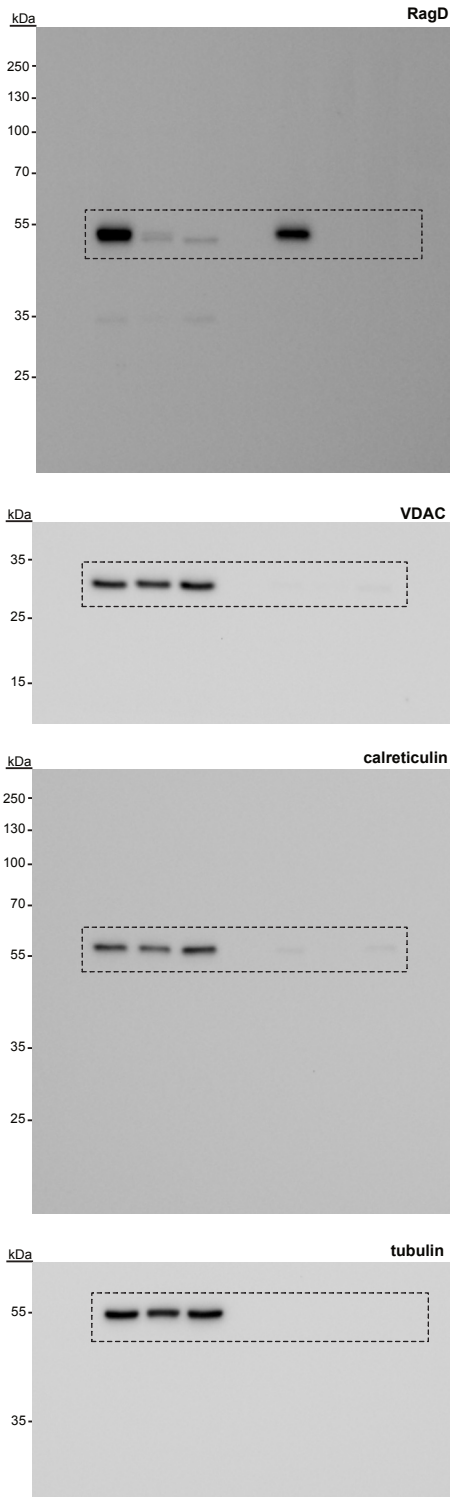

ED Fig. 3I unprocessed blots

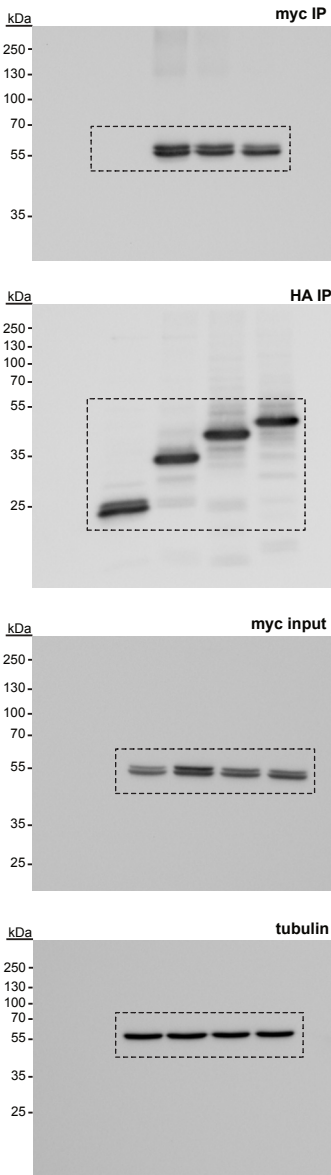

Supplement: Source Data Extended Data Fig. 3 — Unprocessed western blots. [file 41556_2022_977_MOESM20_ESM.pdf]

ED Fig. 4c unprocessed blots

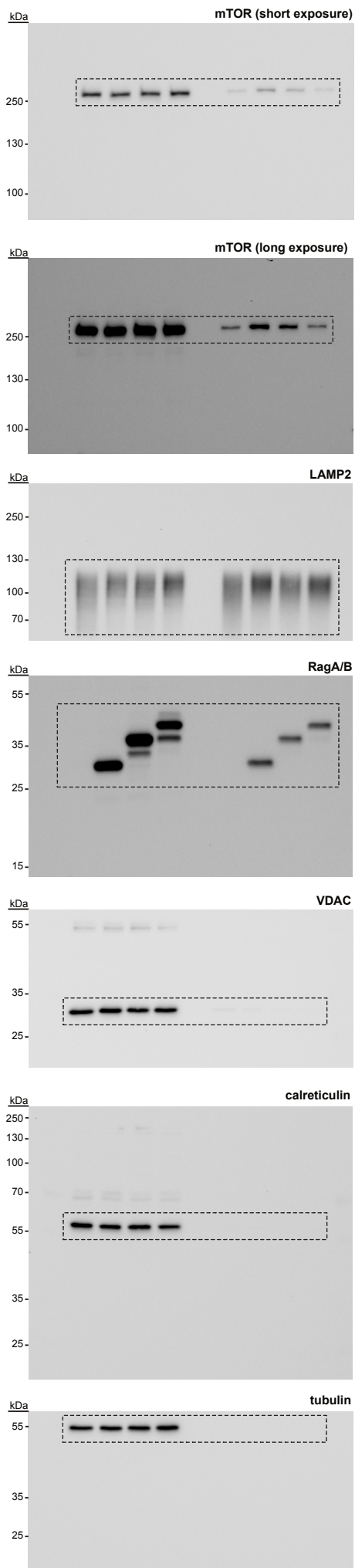

ED Fig. 4e unprocessed blots

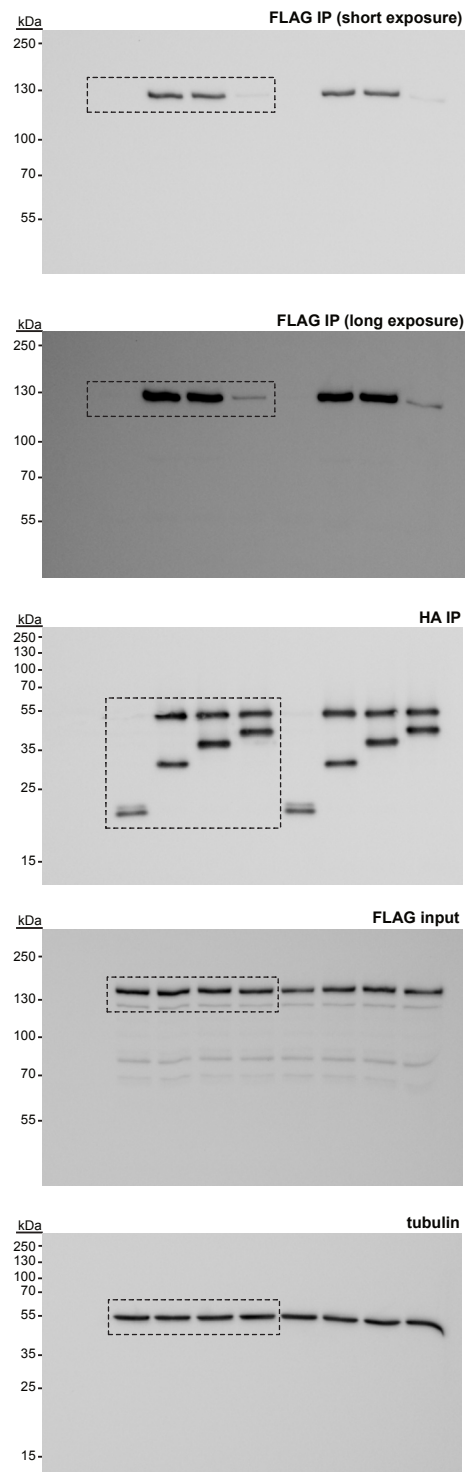

Supplement: Source Data Extended Data Fig. 4 — Unprocessed western blots. [file 41556_2022_977_MOESM22_ESM.pdf]

ED Fig. 5a unprocessed blots

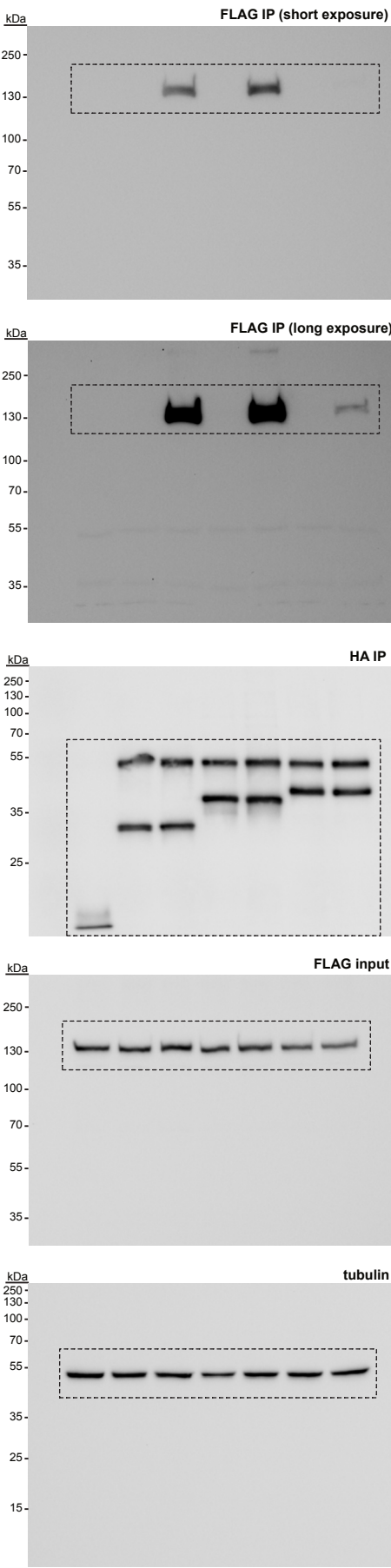

ED Fig. 5c unprocessed blots

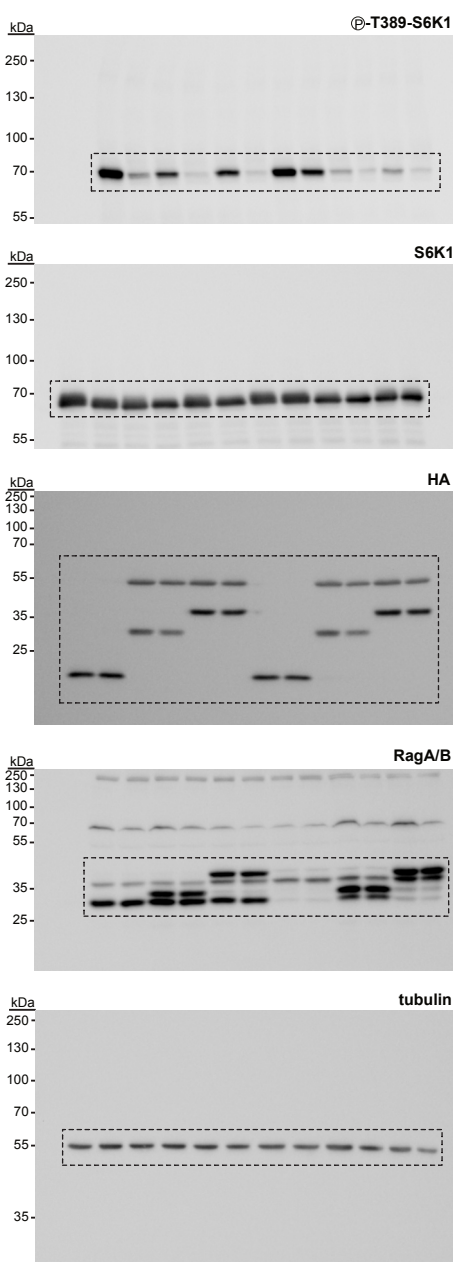

ED Fig. 5e unprocessed blots

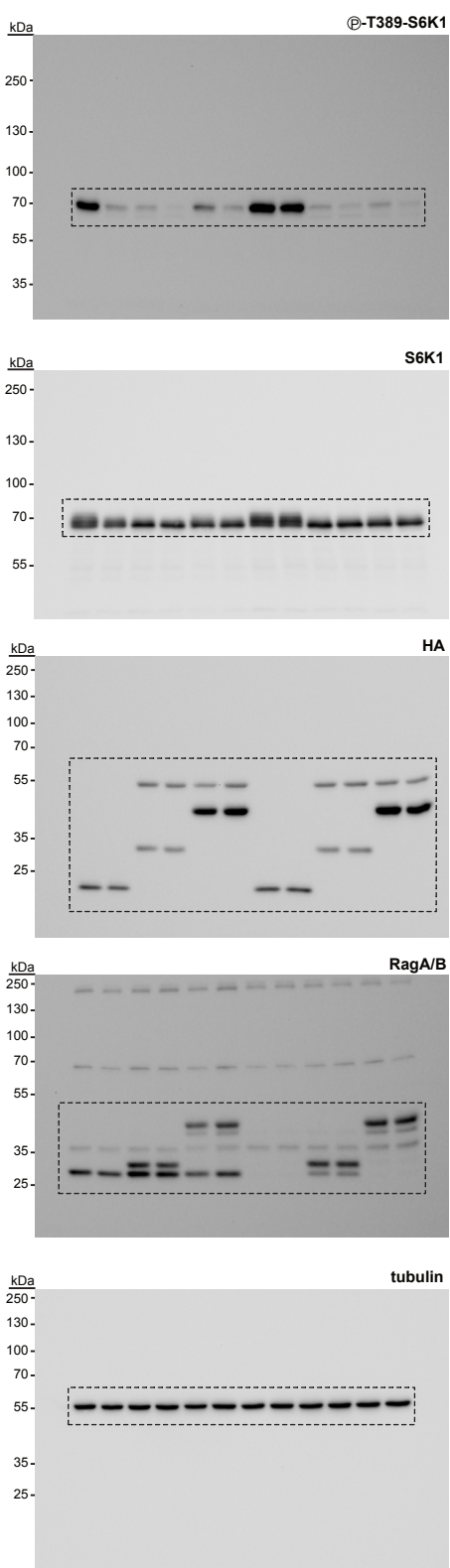

Supplement: Source Data Extended Data Fig. 5 — Unprocessed western blots. [file 41556_2022_977_MOESM24_ESM.pdf]

ED Fig. 10a unprocessed blots

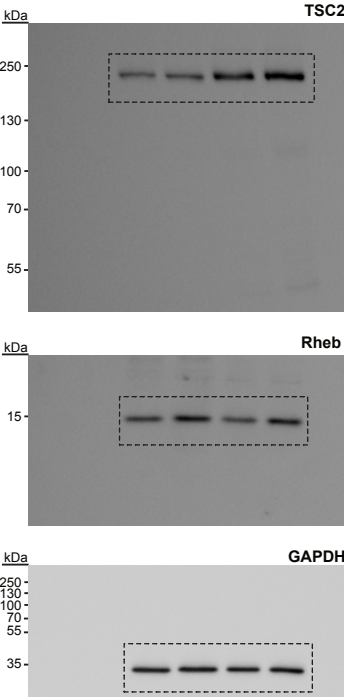

ED Fig. 10b unprocessed blots

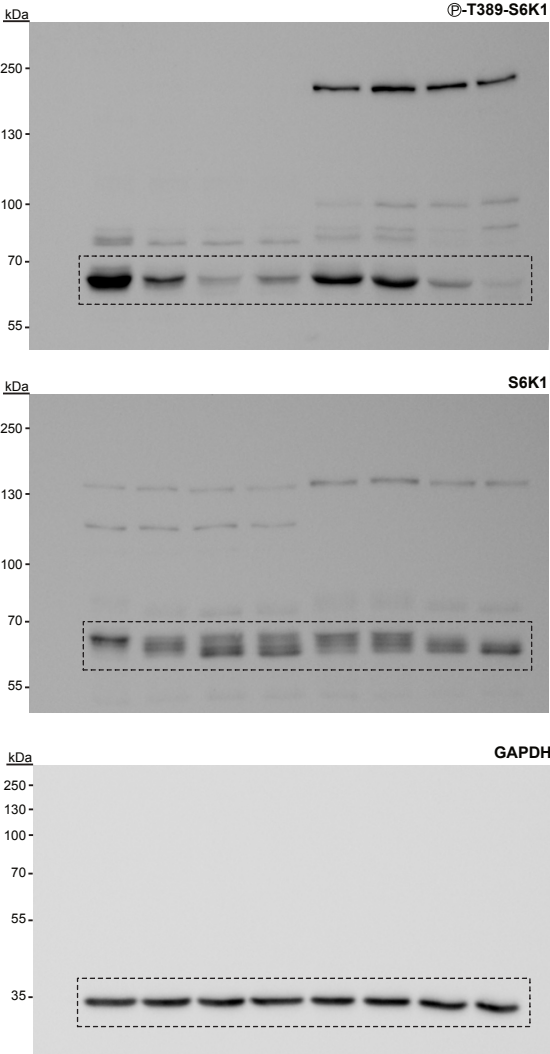

ED Fig. 10d unprocessed blots

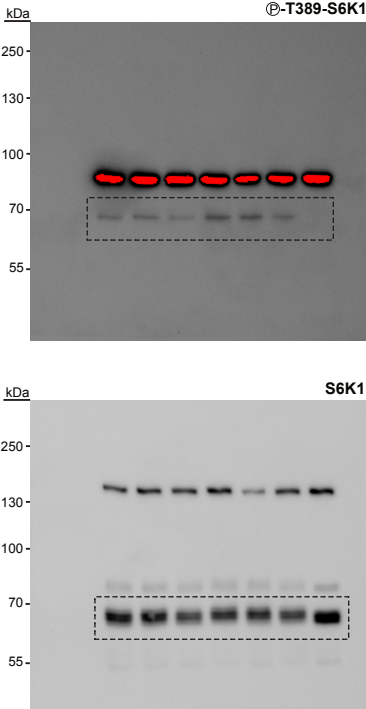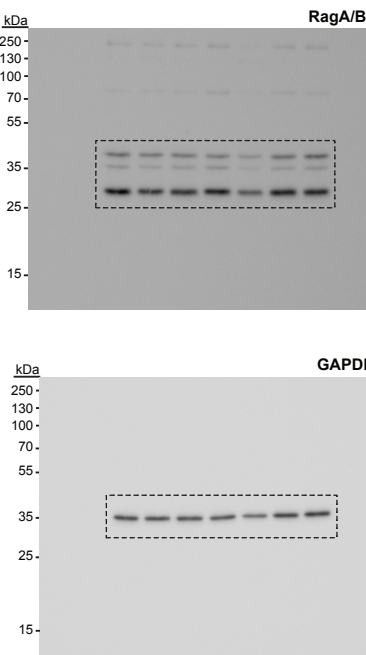

Supplement: Source Data Extended Data Fig. 10 — Unprocessed western blots. [file 41556_2022_977_MOESM33_ESM.pdf]
