# Supplementary material for: Brain-enriched RagB isoforms regulate the dynamics of mTORC1 activity through GATOR1 inhibition
Source: Nat Cell Biol. 2022 Sep 12;24(9):1407–21. doi: 10.1038/s41556-022-00977-x (PMC9481464; doi:10.1038/s41556-022-00977-x)

Fig. 6d unprocessed blots

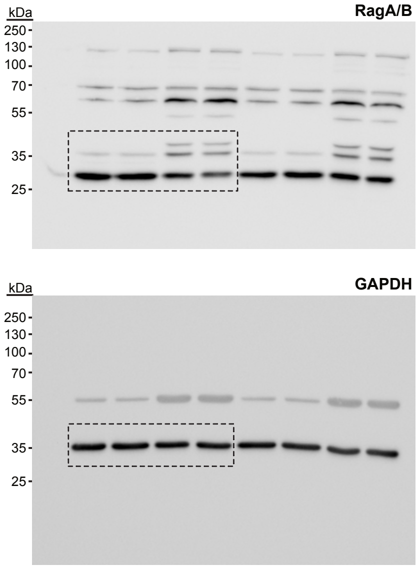

Fig. 6e unprocessed blots

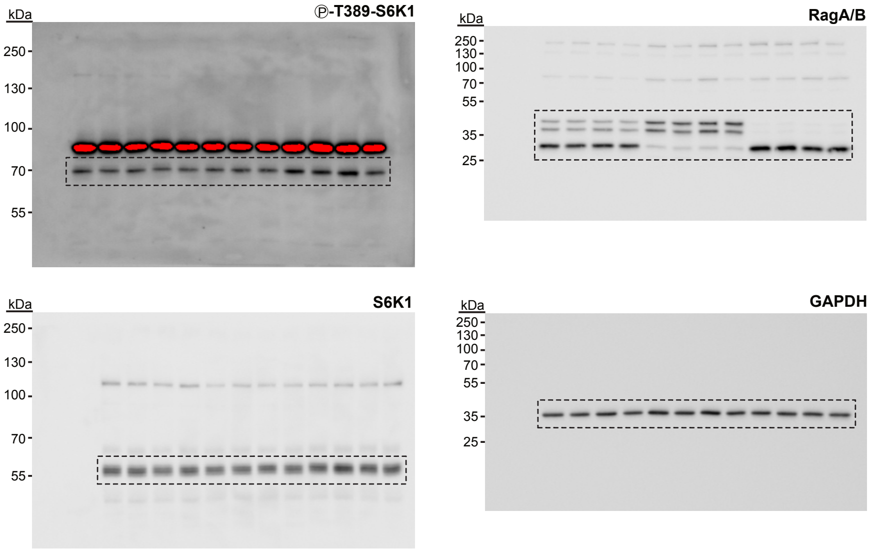

Fig. 6m unprocessed blots

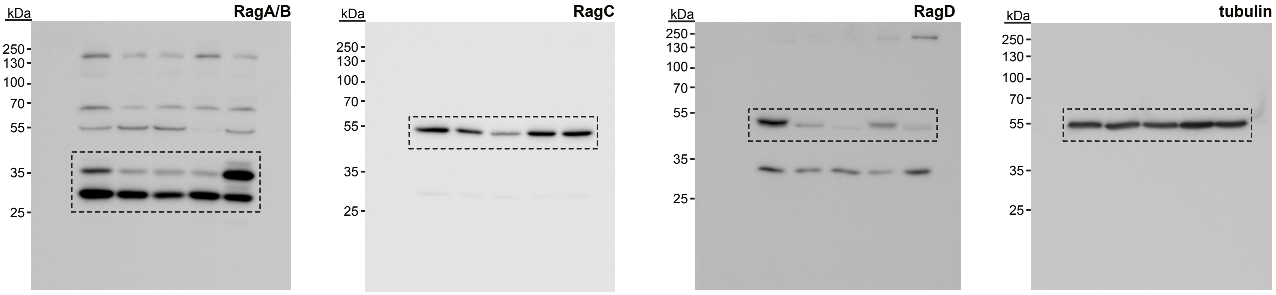

Fig. 6n unprocessed blots

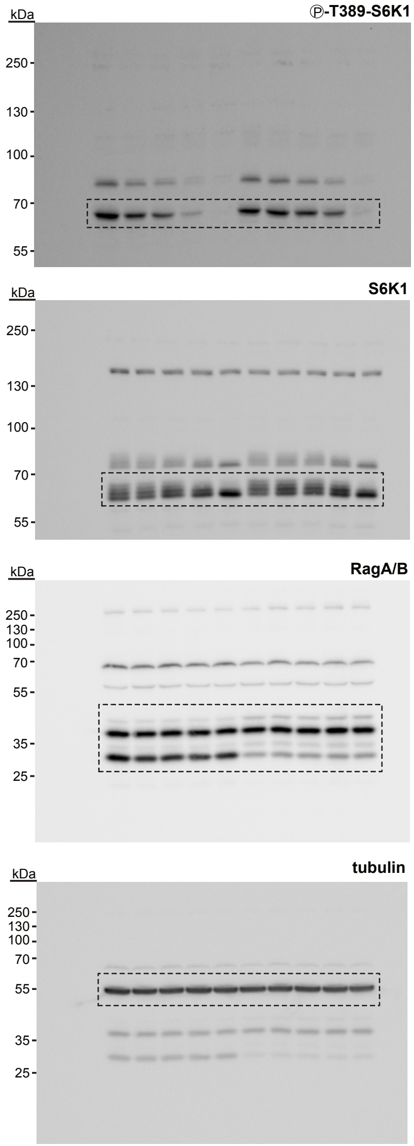

Fig. 6p unprocessed blots

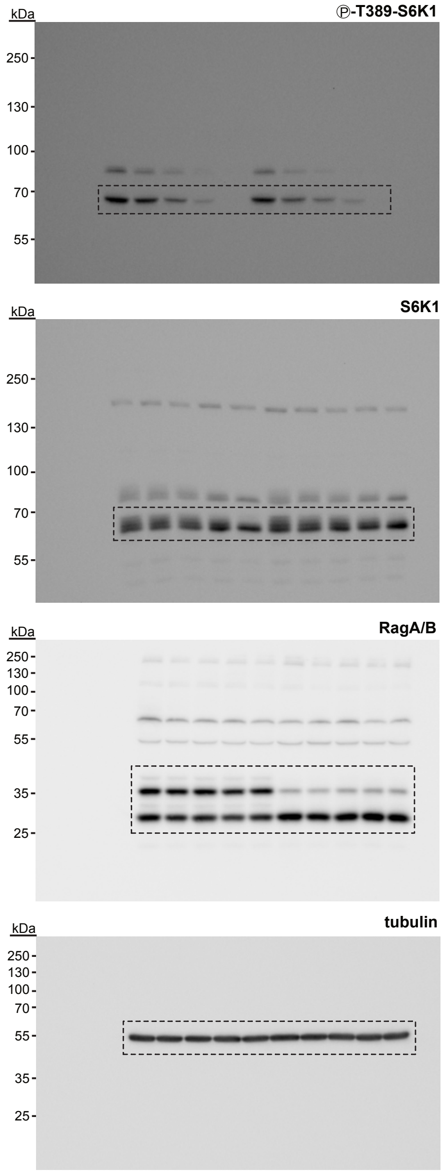

Supplement: Source Data Fig. 6 — Unprocessed western blots. [file 41556_2022_977_MOESM14_ESM.pdf]
