# Supplementary material for: Brain-enriched RagB isoforms regulate the dynamics of mTORC1 activity through GATOR1 inhibition
Source: Nat Cell Biol. 2022 Sep 12;24(9):1407–21. doi: 10.1038/s41556-022-00977-x (PMC9481464; doi:10.1038/s41556-022-00977-x)

ED Fig. 6a unprocessed blots

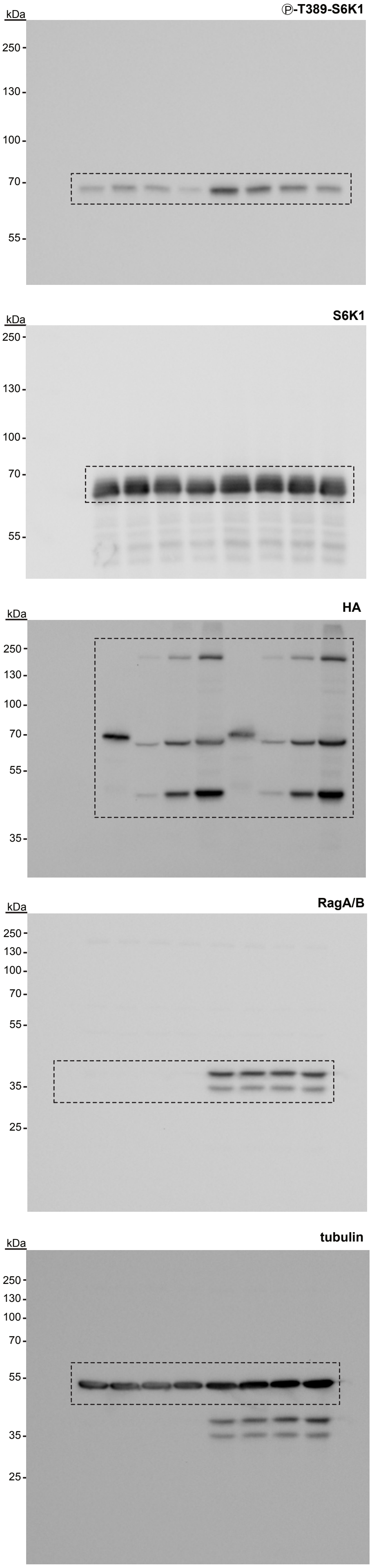

ED Fig. 6c unprocessed blots

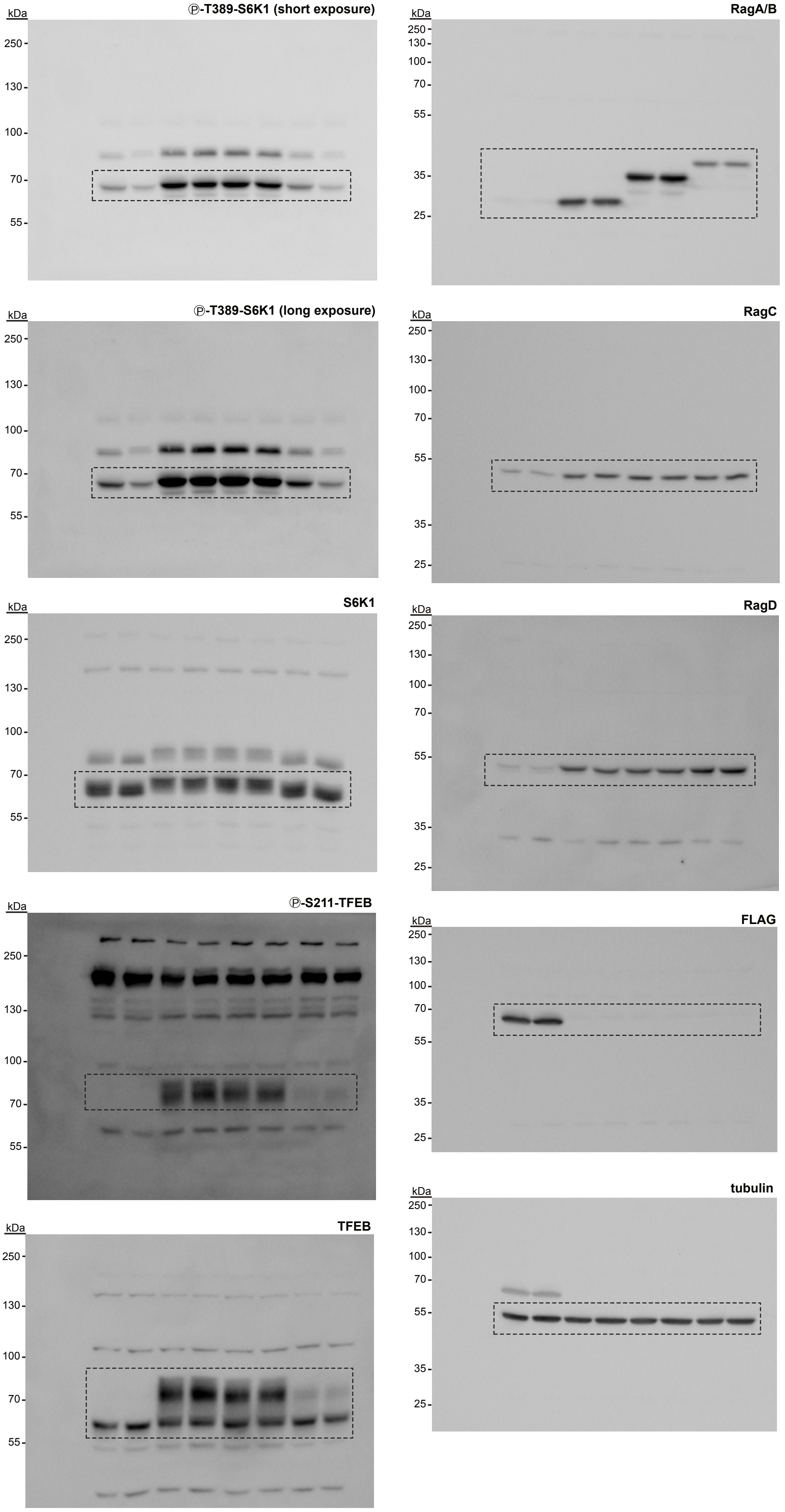

ED Fig. 6g unprocessed blots

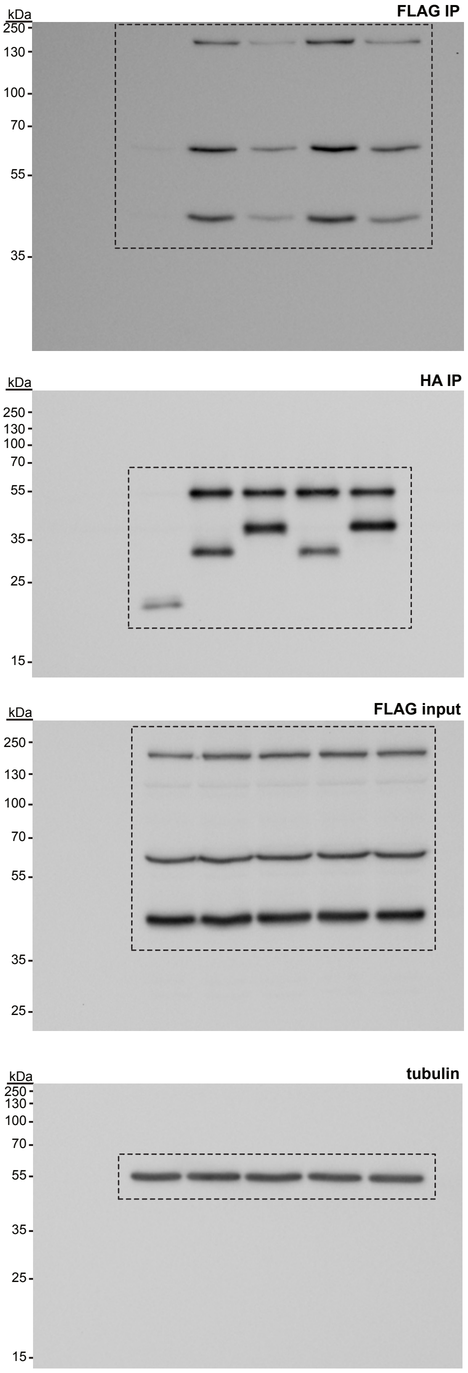

ED Fig. 6i unprocessed blots

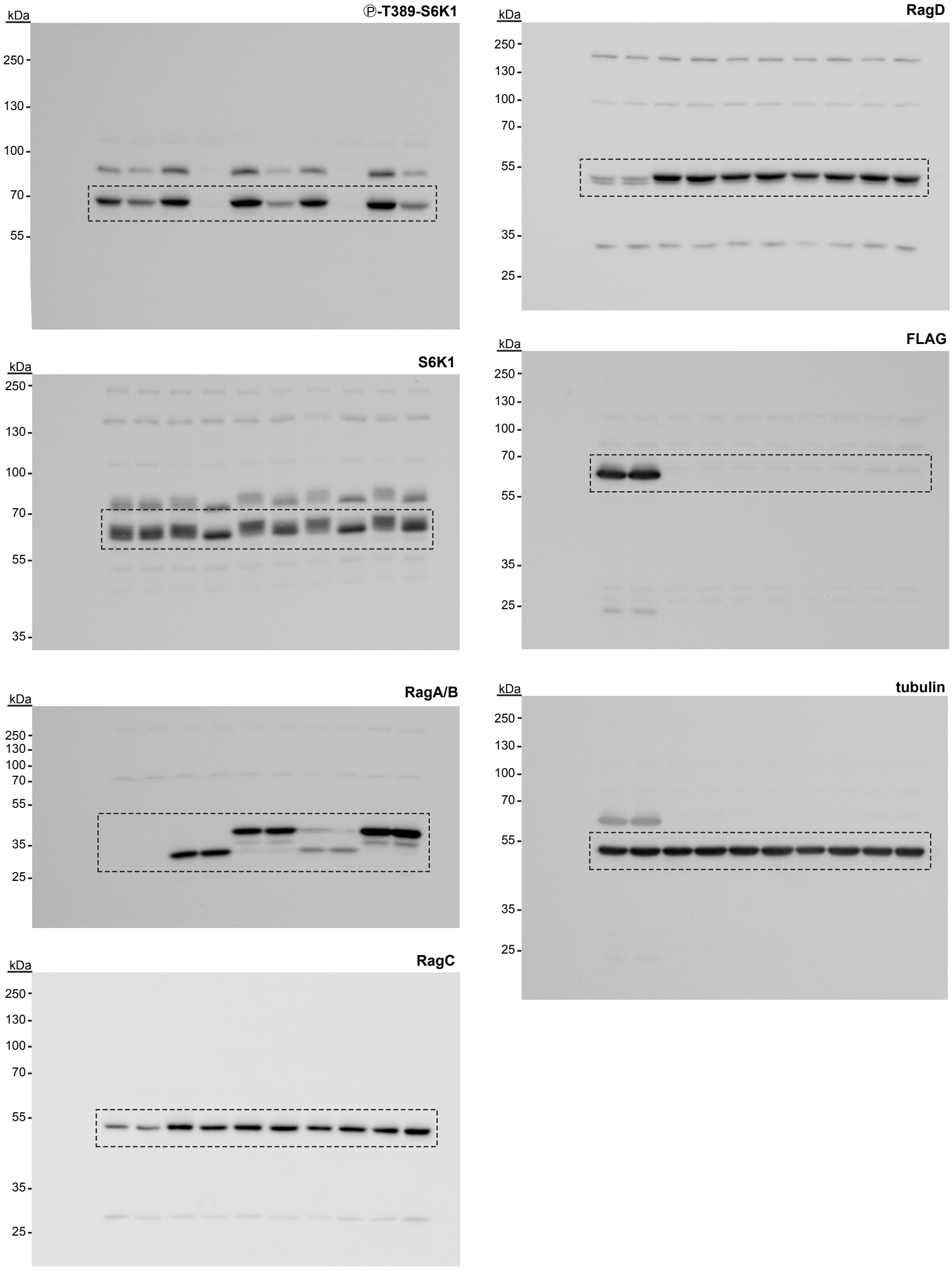

Supplement: Source Data Extended Data Fig. 6 — Unprocessed western blots. [file 41556_2022_977_MOESM26_ESM.pdf]
