# Supplementary material for: Brain-enriched RagB isoforms regulate the dynamics of mTORC1 activity through GATOR1 inhibition
Source: Nat Cell Biol. 2022 Sep 12;24(9):1407–21. doi: 10.1038/s41556-022-00977-x (PMC9481464; doi:10.1038/s41556-022-00977-x)

ED Fig. 8a unprocessed blots

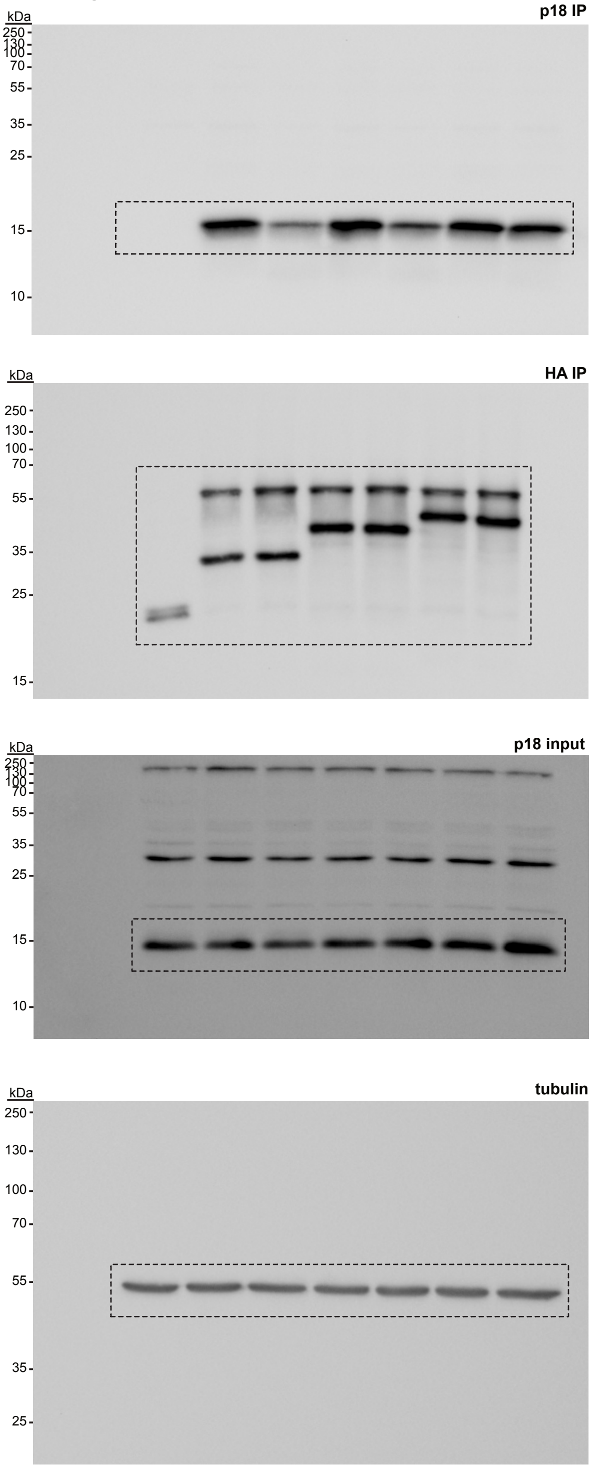

ED Fig. 8c unprocessed blots

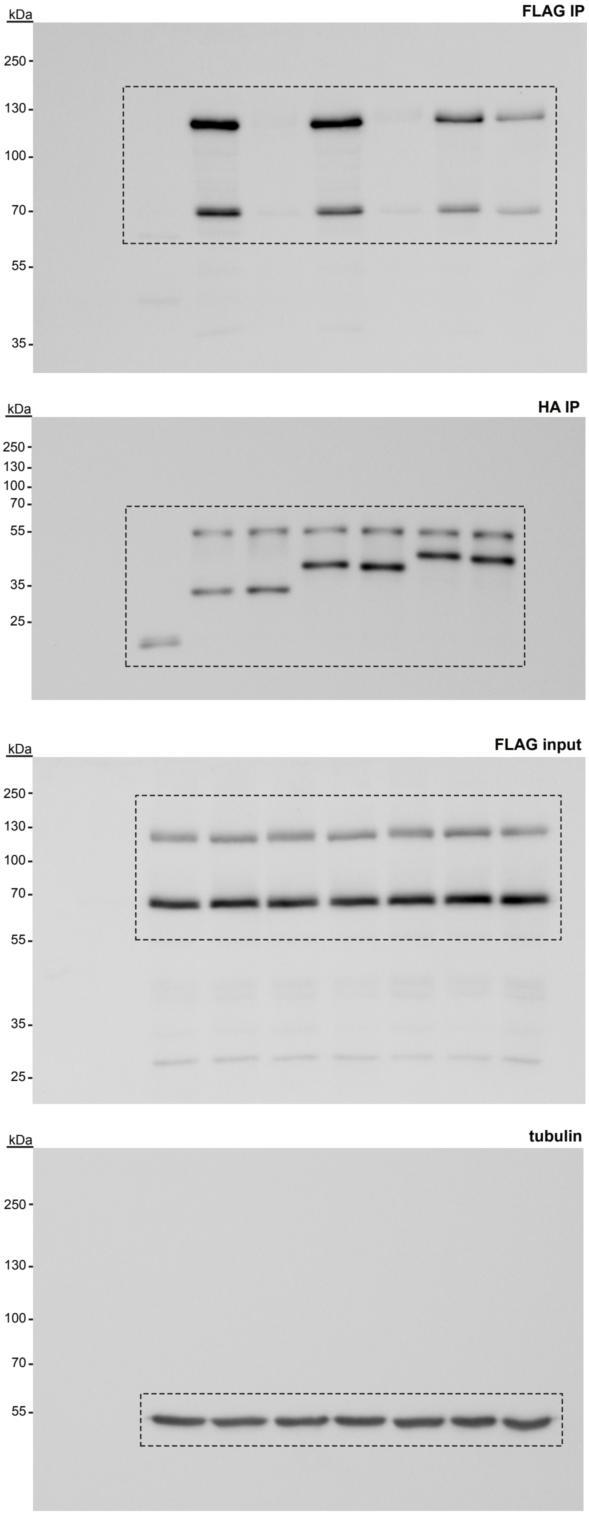

ED Fig. 8e unprocessed blots

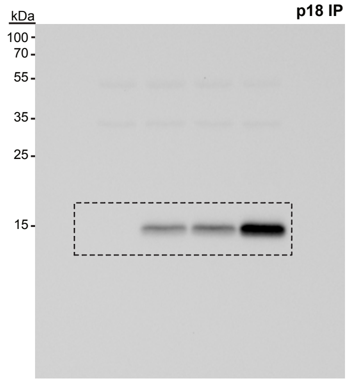

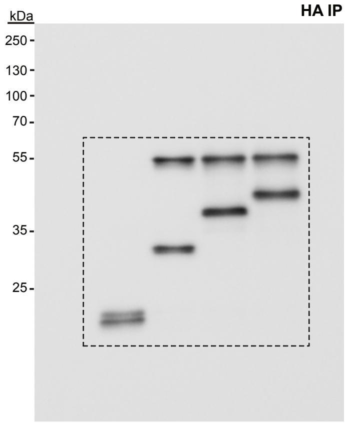

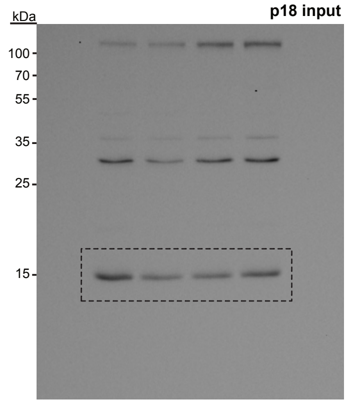

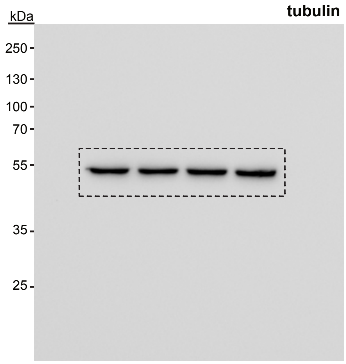

ED Fig. 8g unprocessed blots

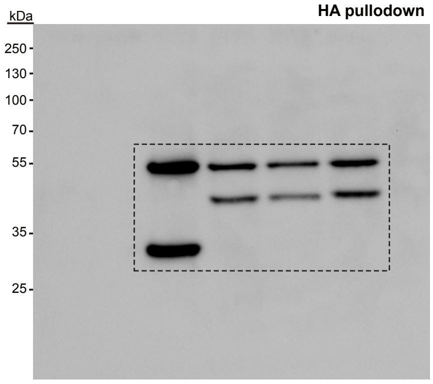

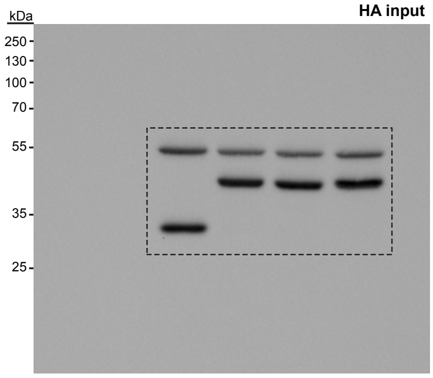

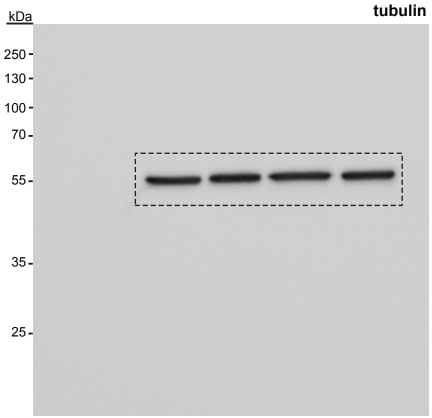

ED Fig. 8i unprocessed blots

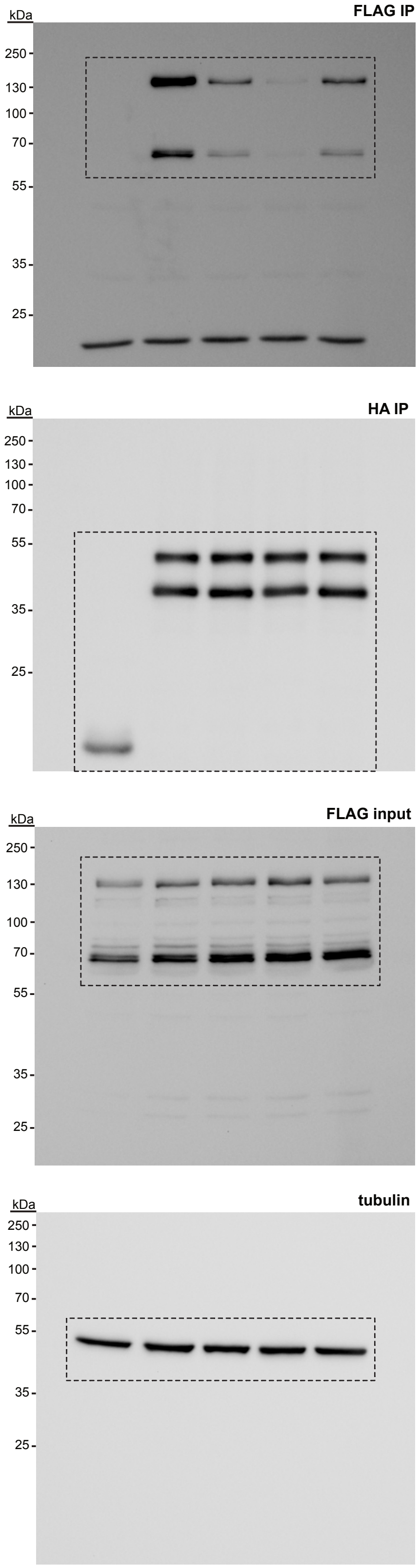

ED Fig. 8k unprocessed blots

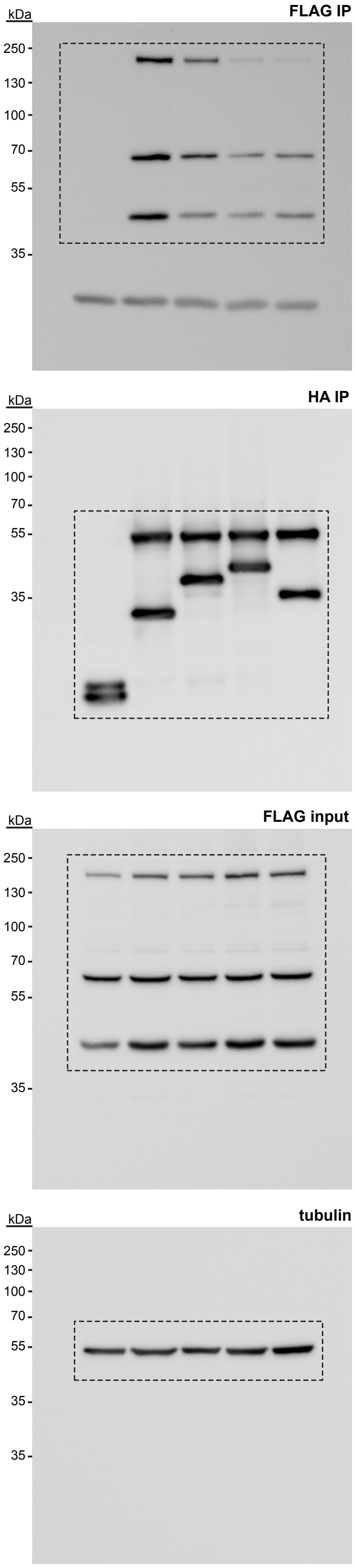

Supplement: Source Data Extended Data Fig. 8 — Unprocessed western blots. [file 41556_2022_977_MOESM29_ESM.pdf]
