# Supplementary material for: Brain-enriched RagB isoforms regulate the dynamics of mTORC1 activity through GATOR1 inhibition
Source: Nat Cell Biol. 2022 Sep 12;24(9):1407–21. doi: 10.1038/s41556-022-00977-x (PMC9481464; doi:10.1038/s41556-022-00977-x)

ED Fig. 9e unprocessed blots

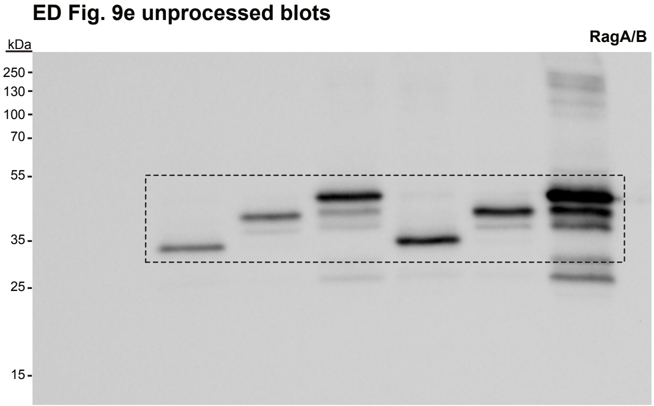

ED Fig. 9h unprocessed blots

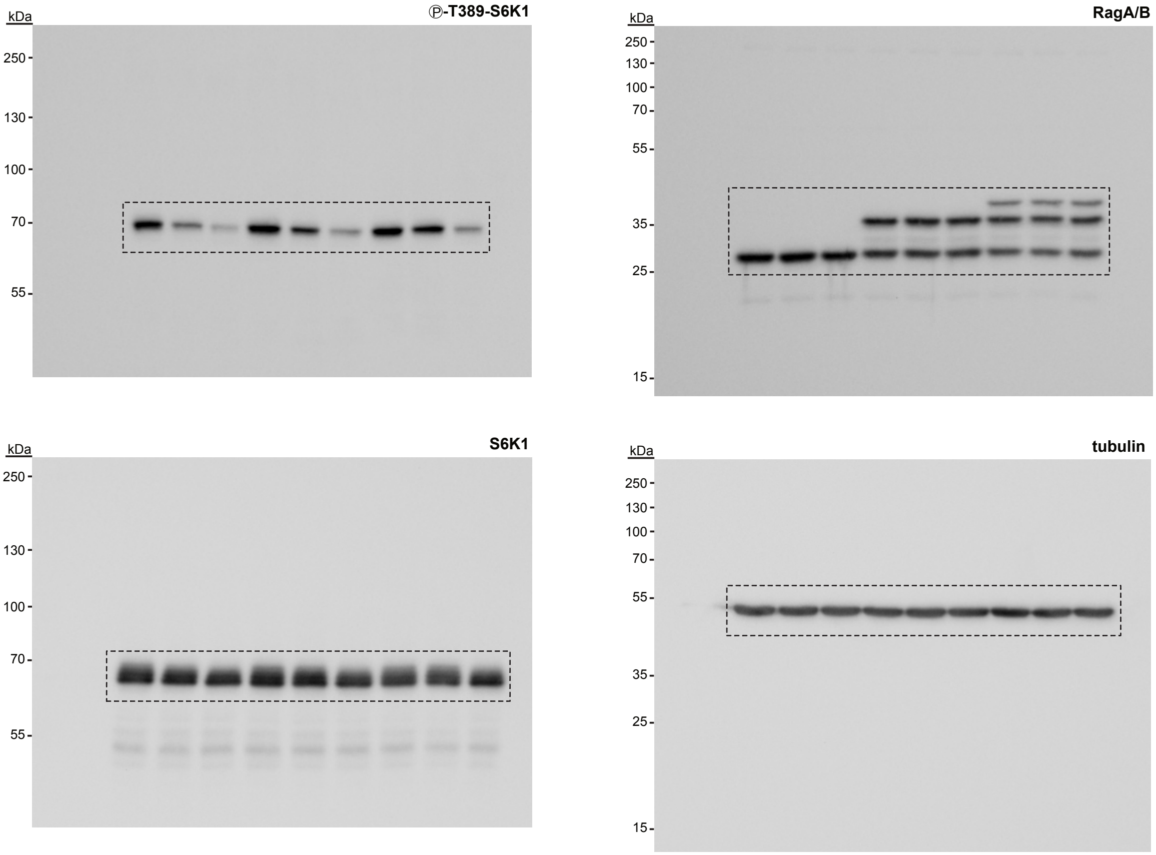

ED Fig. 9j unprocessed blots

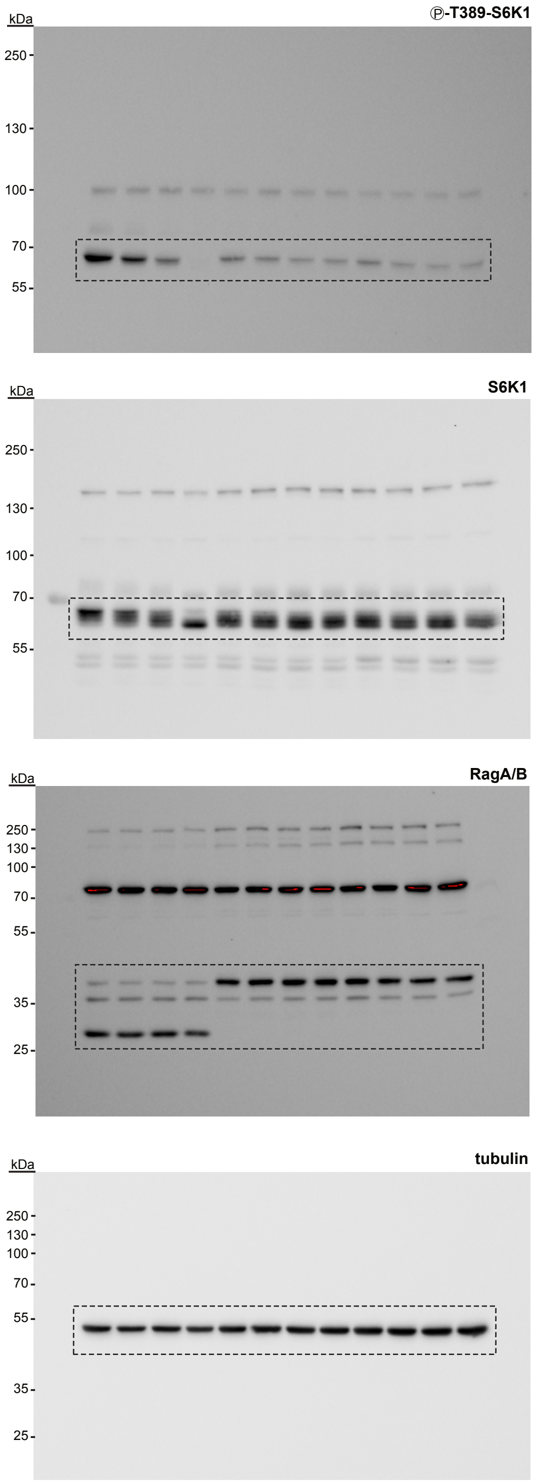

ED Fig. 9l unprocessed blots

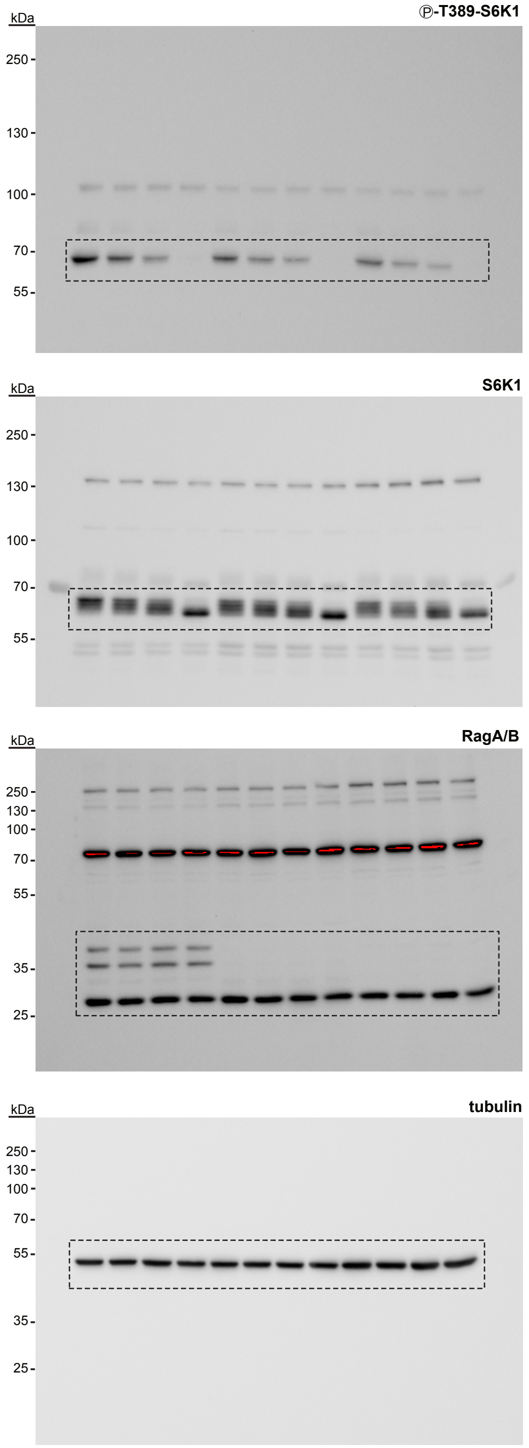

Supplement: Source Data Extended Data Fig. 9 — Unprocessed western blots. [file 41556_2022_977_MOESM31_ESM.pdf]
